# Beyond troubled and untroubled positions – an intersectional analysis of siblings who are bereaved by drug-related deaths’ meaning-making stories about their deceased brothers and sisters

**DOI:** 10.1080/17482631.2024.2372864

**Published:** 2024-06-26

**Authors:** Gunhild Meen, Monika Alvestad Reime, Sari Lindeman, Lillian Bruland Selseng

**Affiliations:** Institute of Welfare and Participation, Western Norway University College of Applied Science, Bergen, Norway

**Keywords:** Siblings, problematic substance use, addiction, drug-related death, discourse psychology, intersection, social category, bereavement, meaning-making

## Abstract

**Purpose:**

This study investigates how social categories work and intersect in siblings bereaved by drug-related deaths’ (DRDs) stories about their relationships to their deceased brother or sister. The sociocultural embedded process of making meaning of the relationship with the deceased individual is essential in adapting to the loss. However, insight into such experiences of siblings bereaved by a DRD is scarce. Previous research has suggested that DRDs may be stigmatized life experiences for bereaved family members, and this paper furthers understanding of the experiences and issues involved in losing a sibling in a stigmatized death.

**Methods:**

An intersectional analysis is applied to interviews with 14 bereaved siblings. By investigating and displaying how different categories intertwine, various positionings are identified.

**Findings:**

Categorization of the deceased siblings as “addicts” constructs a troubled position. However, when “addict” intersects with the categories “unique,” “sibling,” and “uncle,” the troubled subject’s position as an “addict” can be concealed.

**Conclusions:**

Normative conceptions of addiction and DRDs produce troubled subject positions. By intermingling the category of “addict” with other categories, less problematic positions are created. Still, intersections of categories can also construct further complexities of remorse and self-blame for the bereaved siblings.

## Introduction

This article focuses on siblings bereaved by drug-related deaths (DRDs) and the relationships they had with their deceased brothers or sisters with problematic substance use (PSU). Deaths caused by PSU are of international concern (European Monitoring Centre for Drugs and Drug Addiction, 2022), and Norway is among the European countries with the highest recorded number of DRDs per capita (Norwegian Directorate of Health, 2022). There are approximately 10 close bereaved family and friends for every person who dies from an overdose (Norwegian Directorate of Health, 2019), many of whom are siblings. Even though sibling relationships are the longest-lasting relationships most people have, many bereaved siblings experience a lack of social and cultural recognition (Davidson, 2018).

Bereaved siblings have an increased risk of developing mental health challenges (Bolton et al., 2016; Rostila et al., 2019) and of dying an early death themselves (Rostila et al., 2012; Yu et al., 2017). Even though siblings of individuals with PSU are highly affected (Howard et al., 2010), they seem to be poorly acknowledged in research concerning their position as next of kin (Ólafsdóttir et al., 2020; Tsamparli & Frrokaj, 2016), and in their bereavement (Løberg et al., 2022; Perrin, 2023; Templeton et al., 2018).

Research on sibling relationships “as intersecting” with drug use and DRDs is insufficient (Perrin, 2023). Some studies about experiences of PSU in the family have included the sibling perspective (see, e.g., Barnard, 2005, 2007; Lindeman, Lorås, et al., 2023; Ólafsdóttir et al., 2020), but only a few have focused exclusively on the siblings’ relationship experiences with a brother or sister with PSU (see, e.g., Howard et al., 2010; McAlpine, 2013; Tsamparli & Frrokaj, 2016). Howard et al. (2010) show that PSU in a family provokes experiences associated with tension, distress, and loss for family members and can destroy family cohesion and create chaos. In McAlpine’s (2013) doctoral thesis on the experiences of adult siblings of illicit drug users, she found that adult siblings experience distress caused both by their sibling’s problems as well as from witnessing how their parents and other family members are affected. Tsamparli and Frrokaj (2016) reveal how PSU can radically change the quality of siblings’ relationships from being warm and close to causing feelings of anger. Siblings also describe being ashamed and embarrassed by their brothers or sisters with PSU (Barnard, 2007; Tsamparli & Frrokaj, 2016) and experience high levels of stress due to the fear of death of their siblings (Tsamparli & Frrokaj, 2016). Concurrently, Ólafsdóttir et al. (2020) describe the mental health of siblings who are next of kin as less affected than that of other family members, such as parents or children.

### Sibling bereavement after a drug-related death

A few studies about family bereavement after DRDs include the sibling perspective (Bottomley et al., 2023; Dyregrov & Selseng, 2021; Lambert et al., 2022; O’Callaghan et al., 2023; Titlestad & Dyregrov, 2022; Titlestad et al., 2021). However, in most of these articles, the particularities of these are only vaguely described. Nevertheless, it is reasonable to assume that such findings might also apply to the bereaved sibling, as well as to their other family members, if discrepancies between the family members are not specified in the articles. Titlestad et al. (2021) conducted a systematic review of research on family members’ experiences of drug death bereavement that shows that life before death strongly influences the time after death. Another work confirms that family members experience guilt and shame, both before their family member with a PSU dies as well as after their death (Bottomley et al., 2023). Bereaved families also experience stigma from their social surroundings, such as family and friends, work colleagues, and professionals in the public help services, as well as from the media (Dyregrov & Selseng, 2021), and they report relatively high levels of perceived stigmatization (Bottomley et al., 2023; Reime et al., 2024). However, there is a diversity in how the different members of the family experience and deal with the family member’s PSU (Lindeman, Lorås, et al., 2023) and how they cope (Titlestad & Dyregrov, 2022), as well as their experiences of and needs for help and support (Kalsås et al., 2023).

A few studies have investigated siblings bereaved by DRDs, a number of which are derived from the same research project as this article (Dyregrov et al., 2022; Lindeman, Selseng, et al., 2023; Løberg et al., 2022; Lorås et al., 2023). In one paper from Australia, Perrin (2022) explores DRD-bereaved siblings’ experiences and points out how a drug-related death is often a stigmatized death and, therefore, prone to a lack of social and cultural recognition. As a response, the siblings make efforts to protect themselves, their family, and the deceased individual from the consequences of stigmatization. These efforts can range from concealing the cause of death from others to ensuring that friends with problematic substance use do not attend their siblings’ funerals (Perrin, 2022).

There is a need for more research that can expand our understanding of sibling bereavement in the case of DRDs, which includes both the time before death and adaption to the loss. Siblings’ experiences of quality in their relationships with a brother or a sister have significance for how they develop and experience their well-being and relationships with their partner and their social surroundings (Jensen et al., 2023). Bereaved siblings describing their relationships with their deceased siblings as of poor quality are more likely to experience higher levels of prolonged grief compared to those who characterize these relationships as good (Chua, 2020). In addition to the scarcity of studies on bereaved siblings after DRDs, Greif and Woolley (2016) argue that there is a gap in research providing knowledge on how siblings in adulthood are influenced by a brother or sister’s PSU. Howard et al. (2010) point out that there are many different narratives related to individual and relational dimensions of having brothers or sisters with PSU. It is crucial to consider the bereaved siblings’ experiences within their sociocultural context, as the stories told are linked to the norms at stake in specific everyday situations. To better understand sibling relationships, it is essential to explore how siblings classify, connect, and relate to each other. This involves looking at both cultural categorizations that represent the bonds between them, as well as the practical, material, and emotional aspects of their relationship (Gulløv et al., 2021). In this work, we aim to contribute to this knowledge gap by exploring how bereaved siblings, after a DRD, categorize and make meaning of the relationship they had with their deceased brother or sister and how the intersections of categories produce subject positions that are troubled to varying extents.

### Meaning-making by social categorizing

Meaning-making is highlighted as essential in enabling bereaved individuals to adapt to their loss (Neimeyer et al., 2014; Stroebe & Schut, 2010) and “to be able to live on after the loss of a family member” (Titlestad et al., 2021, p. 515). Bereavement is often considered an internal process, but it is also a social one in which bereaved individuals can reorient themselves to practices and everyday activities (Stroebe & Schut, 2010). Furthermore, the meaning-making of the loss is embedded in social and cultural contexts and draws on understandings about bereavement that are available to the bereaved individuals in their sociocultural environment (Thompson et al., 2016).

Categorization is one component of meaning-making. Social categories define individuals by specific characteristics that set them apart from other individuals. As such, these characteristics create distinctions and contrasts among individuals in different social categories. In the social world, boundaries of social groups are formed by social differences, which can be based on assumptions or actual differences in people’s thoughts, emotions, and behaviours. By highlighting significant characteristics, categorization directs attention to contextually relevant and meaningful aspects of the world. When analysing social categorization processes, it is important to consider the social relationships that exist between different categories (Hogg, 2001). Examining how stories of loss are embedded in social and cultural contexts can be achieved by studying how the bereaved siblings categorize their relationship with their deceased brothers or sisters to give meaning to it (Staunæs, 2003).

Social categories select and order people into groups based on specific signs or criteria and are commonly perceived as fixed variables that individuals carry with them without alteration (Staunæs, 2003), such as “male,” “brother,” or “drug addict.” Nevertheless, an individual can be referred to in several ways, such as ’my brother,’ “my late brother,” “my younger brother,” “Thomas,” “Little Tommy,” “a real addict,” “my best friend,” etc. The choice of lingual terms actively shapes meaning by highlighting or obscuring certain aspects of identity, illustrating the significance of understanding how categorization communicates (Hogg, 2001; Housley & Fitzgerald, 2015). The different options for categorizing create different inferences and hold distinct connotations and implications. Specific messages or sentiments are communicated by choosing one term over others (Housley & Fitzgerald, 2015). For example, the use of “my best friend” or “Little Tommy,” expresses endearment, closeness, or a dynamic of responsibility. By employing terms that indicate familial ties, social conventions, and obligations associated with sibling relationships are brought into the conversation. Referring to a brother as “a real addict,” or by his full name, “Thomas,” may convey a distance in the relationship.

There is also a strong association between categorization and stereotypical attributes (Hogg, 2001). Being categorized as a stereotypical “drug addict” may carry deep-seated cultural assumptions regarding the individual’s behaviour and personality and can bear consequences of moral judgement from others due to the stigma associated with the category (Sibley et al., 2023). As such, an investigation into categorizing can explain how positions, hierarchies of power, and mechanisms of inclusion or exclusion may be produced (Staunæs, 2003).

Staunæs claims that “Social categories are not something you are or something you have; rather, social categories are something you do” (Staunæs, 2003, p. 104). Different situations will make varying categories relevant. Therefore, social categories can be depicted as dynamic and contextual (Staunæs, 2003). The understandings of the categories, such as “siblings,” are culturally constructed (Gulløv et al., 2021); for example, the “sibling” category can change over time and place in terms of its content, intensity, and form.

An intersectional perspective highlights the complexity of being positioned in several categories simultaneously (Phoenix, 2006). This concept, as used in this article, investigates categorization as situated and contextual (Orupabo, 2014) and explores how the use of different social categories works together in people’s daily lives and thus in their understanding of themselves and each other (Ulvik & Gulbrandsen, 2019). The value of studying how social categories intersect in concrete and diverse situations is due to the categories’ production of positions with different levels of power and issues (Staunæs, 2003). In this article, we utilize the concept of intersectionality as an analytical tool to explore how different categories work and intersect in the bereaved siblings’ stories about their relationships with their deceased brother or sister and what consequences the intersections have in repairing these siblings’ troubled subject position as “addicts.” Enhancing awareness and deepening insight into the significance of categorization processes can broaden practitioners’ and social affiliates’ comprehension of the sociocultural context’s pivotal role in shaping the experiences of stigmatized bereavement (Sibley et al., 2023).

## Methodology

This work is part of “The Drug Death-Related Bereavement and Recovery Project” (the END project), a large Norwegian cross-sectional, mixed-methods study (2017–2024).

### Participants

Fourteen participants were recruited from a total of 79 bereaved individuals with siblings deceased due to DRDs who responded to a survey administered by the END project. In the informed consent document, they gave their contact information if they were willing to be contacted to be interviewed about their experiences. The 14 participants were selected to ensure variation in terms of gender, age, and place of residence, as well as time since their siblings’ deaths.

### Interviews

Semi-structured in-depth interviews were undertaken in two sections, following two different interview guides (see Table 1): section A (time before death) and section B (time after death). Section A was conducted by three researchers from the END project in 2019 (one of whom is the third author of this article), with all 14 participants involved. The interviews took an overarching, retrospective focus on the siblings’ experiences of the time before death. Section B was conducted by three other researchers from the END project in 2018 and 2019. Ten of the 14 participants were involved and shared their experiences after the death of their sibling. The interview guides focused to various degrees on themes regarding the time before their sibling died and how they helped and supported them, experiences of receiving public help and social support both as next of kin and as bereaved, experiences of stigma, and questions related to post-traumatic growth. All interviews were conducted in Norwegian and transcribed verbatim. Quotes included in the present article have been translated into English by the first author. All participant data are published in a non-identifiable manner by using pseudonyms and changing recognizable details. Some quotations have been shortened by removing filler words and digressions.

**Table I.** Participant information.

Pseudonym	Section	Sex	Age	Deceased sibling	Years since death
Aron	A + B	male	38	older brother	9
Barbara	A	female	44	older brother	30
Catherine	A + B	female	30	older brother	2
Doris	A + B	female	51	older sister	17
Eva	A	female	32	younger brother	5.5
Fanny	A + B	female	36	older brother	1.5
Gloria	A + B	female	35	older brother	7.5
Harriet	A + B	female	41	younger brother	4.5
Iris	A + B	female	45	younger brother	12
Jonathan	A + B	male	37	younger brother	10
Klara	A + B	female	61	younger brother	18
Leon	A + B	male	53	younger brother	12.5
Mathias	A	male	43	older brother	2
Norah	A	female	33	older brother	12

### Ethical considerations

The study was approved by the Norwegian Regional Committees for Medical and Health Research Ethics (reference number 2017/2486/REK vest). Participants provided written, informed consent following the Declaration of Helsinki (The World Medical Association, 2018), during which they were informed about the work’s purpose, method, and procedures. The transcripts of the interviews were anonymized and securely stored on a research server.

### Limitations and strengths

The interviewers followed the same interview guide linked to the specific interview section (A or B). However, a difference in professional backgrounds and interviewing experiences contributed to some diversity in how the topics were explored. The duration of the interviews, ranging from 45 minutes to three hours, may have been influenced by variations in interviewing style. This divergence in time can be attributed to interviewers either strictly adhering to the interview guide or taking the flexibility to ask follow-up questions. Factors such as requests for breaks, along with the varying levels of detail and expansiveness in participants’ responses, also contributed to the disparate lengths of their narratives. However, it is important to note that the duration of the interviews did not necessarily impact the richness of the data material for this analysis.

The diversity and differences concerning the interviews strengthened the data material by offering a broad range of experiences among bereaved individuals, considering the variations regarding the number of years since the loss and the age of the participants at the time of their bereavement. This variety proved to be valuable for conducting an intersectional analysis. Even though recall bias must be considered, this diversity of retrospective focus is regarded as a strength. The participants’ use of different narratives led by the interview guide and the differences in the interviewers’ follow-up questions contribute to strengthening the data material with both breadth and differences. In addition, analysing both interview sections helped validate the relevance of the categories presented by confirming the extent of their usage.

Still, during the interview process, certain factors may have influenced the participants categorization processes. The participants who were recruited for this study were labelled as “bereaved siblings after drug-related deaths” in the invitation to join the larger survey. During the interviews, the interviewers participated in the categorization process through the ways they referred to the deceased, the interviewed bereaved, and other bereaved individuals.

Additionally, the interview guide’s questions regarding the support the participants had offered their siblings prior to their death, and about the emotional strains resulting from the death, communicate expectations of specific actions and emotional responses.

### Analytical framework and procedure: an intersectional perspective

The analytical approach was inspired by Staunæs (2003), who recommends a focus on the concrete doing and intermingling of categories “where concrete intersections, hierarchies and elaboration are not predetermined” (Staunæs, 2003, p. 102). We opted for an intersectional perspective in our analysis of the data due to the diversity in which the siblings represented their deceased kin throughout the interviews. These individuals commonly navigated through a range of social categories to characterize their siblings, often oscillating between portraying them as a “drug addict,” as ’unique,’ or as their “sibling.” This diversity in portrayals underscored the complex interplay of subject positions, thereby making intersectional analysis a particularly apt and insightful analytical approach to adopt in this context.

Staunæs and Søndergaard (2006) describe this way of using intersectionality in the form of an analytical perspective as a curtain drapery. All social categories a person adheres to are layered, but some are more visible and overshadow others, depending on how the different layers are focused on in varying contexts. Focusing on the complexity and changing nature of lived experience, the concepts of “subject position” and “troubled subject position” are appropriate.

The *subject position* embraces the complexity of how people position themselves and others in their everyday events. Discursive interaction provides a diversity of possibilities of subject positions, which people take up, ignore or resist, or make their own (Davies & Harré, 1990). Such positioning is an ongoing process and depends on what discourses are available and what practices are appropriate, as well as who is involved and how power is distributed (Staunæs, 2003). The concept of *troubled subject positions* refers to positions challenging normativity. These include positions that become inappropriate or difficult because they diverge from the discourse’s normative expectations (Staunæs, 2003).

To become familiar with the data concerning the participants’ categorizations used to make meaning of their relationships with their deceased siblings, the first author started by reading the transcriptions and listening to audio files of the interviews. In line with Staunæs’s (2003) recommendation, we did not look for predetermined categories but explored what appeared in the data. Considerable extracts from the interviews that contained participants’ descriptions of their siblings and sibling relationships were selected, and after sorting the data by such descriptions, categorizations were highlighted. In this phase, we identified noun phrases used to refer to their deceased sibling, such as “my brother,” “a drug addict”, or “uncle.” We also identified categorizing by adjective phrases using words like “unique,” “normal,” “older,” “younger” etc. Moreover, we identified statements describing the sibling by categorization such as “a typical drug addict” or “not such kind of drug addict,” Additionally, we analysed phrases in which the participants related themselves to their siblings, like “I was a very little sister.” Such identified phrases were highlighted and compared to find similarities and differences among them.

Furthermore, we explored how various categories within the narratives were intertwined. We identified how the categorizations could be perceived as troubled positions related to how they diverged from or challenged normative expectations. We found the category of “addict” to be a troubled position that was not adequate for the participants in describing their brother or sister. The analysis showed a pattern where the category of the “addict” was intertwined with other ones. This intersection between “addict” and other categories seemed to seek to understand the complexity of their relations and is interpreted as an attempt to reduce the stigma associated with the category of “addict.”

In the next round of analysis, the first, third, and fourth authors looked more closely at other categories intersecting with the “addict” one. This stage provided a deeper understanding of how the interplay between different categories created subject positions of more or less trouble. When these categories were intermingled, the authors identified how they co-constituted other subject positions that were troubled to varying extents. Guided by the research question, some categorizations were identified as repairing “addict” as a troubled position. For instance, we observed how the intersections with more positively loaded descriptions and categories, such as “he read a lot” or “he took on such a big brother role,” produced a less troubled subject position.

Finally, we named the social categories identified. The names are given to represent portrayals of sibling relations and to show the variations of the most frequent categorizing used by several of the participants.

## Findings

In the following section, the most vital categories used and how they intermingled with the addict category are presented. Most participants categorize their siblings as “addicts” in their portrayals of the relationships they had. This is identified as a troubled position due to the stereotypical understanding of an “addict,” which is a subject position viewed as inappropriate and not culturally acceptable. The category of “addict” is presented as insufficient to fully encompass the complex lived experience of having had a brother or sister with PSU. The participants employed other categories, such as “unique,” “brother” or “sister,” and “uncle”, which served to conceal the troubled position of “addict.” In addition, the findings showed that the intersection of categories may produce new troubled subject positions for both the deceased siblings and the participants and, therefore, cause more possible complexities regarding the participants’ meaning-making of the relationship.

### He was an addict, but also unique

Many participants make meaning of the relationship to their deceased siblings by emphasizing them as “unique,” as a person who had been significant to them in their lives. By portraying their sibling as “unique,” the participants created a dividing line between their sibling and the stereotypical addict. Several stated that it was vital for them that their sibling was understood as an individual and not just as an “addict.” For example, Jonathan reasoned this as follows:

This isn’t a story that’s just, “I had a brother, and then he became a drug addict, and then he died.” He wasn’t the sort of drug addict you see on the street. Many probably say the same, but my brother was unique. He wasn’t one of them. I need to have the opportunity to tell people about him, who he really was, rather than him being reduced to an overdose death.

By portraying his brother as unique and not as “one of them,” Jonathan resisted his brother from being reduced to a drug addict and an overdose death. As such, he overshadowed the categorization of his brother as an “addict.” Categorizing the deceased siblings as “unique” can be interpreted as a strategy through which the participants try to repair the troubled subject position of their brother or sister as an “addict.”

In most of the interviews, the participants used a multitude of qualities to portray their deceased brothers and sisters as unique and special. Words such as smart, kind, skilled, funny, and resourceful were frequently utilized by many of the participants to show how their deceased siblings differed from the stereotypical subject position of “addict.” One example is found in the interview with Klara, who described her older brother, saying, “He was a really great guy. He was smart, he read a lot, and he had lots of resources. But all this was drowned out by his substance use.” Klara’s description gives an impression of her brother as “unique,” and as such, she repaired her brother’s troubled subject position as an “addict.” However, Klara did not obscure her brother’s “addict” side; instead, she made visible the intersection of how her brother as an “addict” finally overshadowed and “drowned out” her brother as “unique” when he was alive.

Most participants portrayed their deceased siblings as diverging from the disadvantageous and negative characterizations of the “addict” stereotype. Gloria, for instance, said about her younger brother: “I was never afraid of him. He was never dangerous or violent or anything like that.” Gloria rejected the idea that he adhered to the characterization of an “addict” as violent. Similarly, in the interview with Mathias, he positioned his older brother as divergent from the stereotype by drawing on his number of accomplishments:

He dabbled in heavy drugs at times, but he was never the kind of junkie who’s been on the street. He had real jobs and always had an apartment. He got a girlfriend, and he had kids. He was a master at keeping his head above water.

Mathias categorized his brother as an “addict” by confirming his use of heavy substances and referring to him as a “kind of junkie.” Moreover, Mathias differentiated his brother from the stereotype by showing him as “unique” and portraying him with the skills of being a “master” of keeping his head above water. Mathias deconstructed his brother’s position as an “addict” by highlighting his culturally valuable activities, including real jobs, apartments, girlfriends, and children, and noting that his brother had never been on the street and, as such, makes his brother’s position less troubled. Gloria’s and Mathias’ categorizations of their deceased brothers as having been “unique” reduced and repaired a troubled subject position related to being an addict. Nevertheless, they also reproduced the stereotype of an “addict” with undesirable characteristics, such as being “violent,” not having a real job, and being on the street.

Furthermore, the findings show how the participants portrayed their siblings as remarkably talented and unique from any “normal” person. Fanny opposed the categorization of her brother as an “addict” by stating that he was so well functioning that “one would never have guessed” he was an “addict.” As opposed to Fanny, Aron promoted his brother’s “addict” side by relating his brother’s great intellect as a reason for him using drugs, saying: “My brother was such a big brain, such an international capability. Artists often have a dark side, which often involves drug use.” Aron’s description of his brother, employing expressions such as “great mind,” “international capacity,” and “artist,” positioned his brother as unique and with valuable qualifications. In this account, Aron also suggested that his brother’s life may be linked to how artists often have a dark side, which many times involves drug use. By doing so, he connected his brother’s life to a group of people and to a lifestyle that is more culturally accepted. Instead of confirming the “addict” as merely a troubled subject position, Aron negotiated the issue of the “addict” position. He somewhat legitimized it by categorizing his “addict” brother within the same “unique” category as those with great minds. As such, Aron reconstructed and offered a new and more valued perspective of the “addict” category, which was helpful in repairing his brother’s positioning as less troubled.

### He was my brother, but also an addict

In the participants’ stories about how they related to their siblings as “addicts,” most participants used categories of “sibling,” brother,’ or “sister” to describe a mutual bond built from a shared childhood and adolescence, having grown up in the same family and being there for each other no matter what. One example appears in the interview with Jonathan describing the relationship he had with his younger brother:

There’s something about that sibling bond, right? Because there’s no other person I’ve gotten so close to, like friends or others in the family. It was me and my brother, we took each other entirely for granted. So, from a very young age, we played together. I looked after him when needed, and he was there for me, you know. It was the two of us. It was never an option to shut the door on him or to say no when he called or asked for something. I just thought, “Of course, I’ll be there; he’s my brother.” I think it was that simple.

Jonathan portrayed their connection as “siblings” as a close bond and different from any other relationship, established by its length and their shared experiences, as well as holding a mutual responsibility for each other. Like Jonathan, many of the participants explained why they supported their siblings by merely referring to them as “siblings.” Jonathan also drew on the categorization of “brother” to justify his actions of not rejecting his “addict” brother.

Several of the participants also used troubled characterizations in the portrayal of their siblings, such as how the sibling was “not reliable,” caused them many disappointments, and could make the participants “embarrassed.” In Mathias’ conversation with the interviewer about the relationship he had with his brother, he made the intersection explicit by categorizing his brother within the distinct categories’ “addict” and “brother”:

Mathias: He was utterly annoying when he was under the influence. No one wanted anything to do with him then, but normally, he was a really all right fellow.

Interviewer: *Is that why you’ve coped to support him as you have because you know who he really is?*

Mathias: Yes. And because he was my brother, and I loved him no matter what.

Mathias confirmed his brother, when positioned as an “addict,” as troubled by displaying the consequence of nobody wanting to have anything to do with him. Nevertheless, he also repaired this troubled position by describing his brother as “really all right,” in contrast to being “annoying.” Furthermore, the interviewer’s question confirmed the troubled position of an “addict” as someone nobody wants to have anything to do with by suggesting that Mathias’s ability to cope with supporting his brother was driven by his position as a “sibling.” In Mathias’s confirmation of this, he explained his supportive actions by implying that being a “brother” overshadowed the subject position of being an “addict” and made him love his brother no matter what.

Some of the participants stated that they regretted their actions towards their deceased sibling. A number of them were remorseful for having argued with their siblings instead of supporting them. Others talked about not having tried to maintain more contact with their siblings. Harriet showed one example of such a regret, saying:

I wish someone had told me that addiction is a disease. Had I known from the beginning, I would have been able to give my brother a completely different sisterly experience than what he got, and I wouldn’t have been so judgmental.

Harriet’s wish that she could have given her brother a “different sisterly experience” can be interpreted as regret and that she wanted to be a “sister” to a greater extent, such as having had a closer relationship and being more supportive and caring. Still, Harriet legitimated her attitude towards her brother before he died by her lack of knowledge of her brother’s PSU as a disease. As such, she displayed two distinct understandings of the term “addict.” When her brother was alive, Harriet held an understanding of an “addict” as a troubled subject position. Her judgemental attitude towards him can be interpreted as Harriet assuming that her brother’s PSU was self-inflicted; thus, her empathy was not triggered. After he died, Harriet had internalized an understanding of being an “addict” as having a disease, and thereby, she positioned her brother as in need of help and support. Even though Harriet was not aware of the understanding of addiction as a disease when her brother was alive, she still regretted her lack of empathy towards him after his death.

The intersection between “addict” and “sibling” has some further consequences. In the participants’ descriptions of their relationships with their deceased siblings, many of the participants gave meaning to the sibling relationships by using categorizations based on birth order. Such categorizations displayed distinctions of positionings in the “sibling” category. Older siblings were discursively constituted as role models for their younger siblings, with a duty of responsibility towards them. Younger siblings were categorized as more vulnerable, with less responsibility, and with the rights to care and protection from the older sibling. Examples of such categorization of sibling responsibilities based on birth order are found in many of the interviews. The discussion with Barbara, who lost her older brother when she was 14 years old, illustrated how her brother’s position as her “big brother” intersected with the “addict” category and caused complications. Barbara described how she met her older brother downtown because of her mother’s restrictions on letting him into their house. Sometimes she wanted to buy him some food because she was concerned for him as an “addict.” However, his position as her older brother made this problematic, and Barbara explains this resistance through their sibling dynamics: “He took on such a big brother role that made it hard for me to care for him.” Barbara’s story shows how normative expectations inherent in the responsibilities of being a “big brother” caused difficulties for her brother to accept help from his “little sister” and for Barbara not to be able to care for her “big brother” as she wished.

### He was my brother, but also uncle to my children

The final intersection presented is how the categories of “addict” are intertwined with those of “sibling” and “uncle.” The participants who had children often categorized their deceased siblings as “uncles” when they gave meaning to their relationships with their siblings in stories involving their children. “Uncle” is used because most of the deceased siblings were men. Most of the participants said that they were preoccupied with maintaining a good relationship between their siblings and their children. The categorization of an “Uncle” was characterized by many siblings by utilizing words such as “funny” and “joyful” and displayed as a positive contribution to the participants’ children’s lives, as noted in the interview with Klara: “Their uncle always held a special place with them. They were very fond of him. We visited him, drove him to a farm, we had a lot to do with each other.”

In addition, the categorizing of their siblings as “uncles” was used by several of the participants to characterize their sibling as a part of their family after their death as well, such as Aron, who said:

I have two children myself. They’re very occupied with the uncle they had. They ask a lot, like most kids do. We normally talk about my brother and laugh at the things that he said and did. But we’re not talking about the painful period leading up to his death.

Aron’s story demonstrates how he positioned his deceased brother to his children as their “uncle” by talking about him and telling funny stories. By describing how he avoided talking about the difficult times, Aron consciously overshadowed the troubled position of his brother as an “addict” towards his children. Nevertheless, a consequence of concealing the “addict” might result in unanswered questions for the bereaved nephews and nieces.

In addition, the participants showed a troubled subject position for themselves in the intersection of categorizing themselves as a “parent” in addition to being a “sibling.” Being a “parent” is characterized by having a diversity of caring responsibilities for children. When the participants categorized themselves as parents, they concurrently justified why they could not be there for their siblings as much as before. Most participants who became parents when their siblings were still alive drew on this categorization as a turning point which changed their capability to fulfil their responsibilities as “siblings.” Klara explained her priorities in placing her children before her brother by categorizing herself as a “parent,” saying; “Then I had children myself, and I couldn’t save the whole world. I had more than enough to make my everyday life go up,” justifying that her responsibilities of becoming a “parent” overshadowed her responsibilities of being a “sister.”

The findings show how the intermingling of the categories “sibling” and “parent” produced troubled positions for the bereaved siblings. Notably, the participants’ desire to conceal the “uncle’s” troubled subject position as an “addict” from the children contributed to causing issues. For example, the participants found it difficult to unify their responsibility as a parent with that of being caring and responsible towards their sibling, as demonstrated in the interview with Leon:

We had a little boy at home and were expecting another one at that time. So, we lived our family life in a small apartment. And even though I wanted to include my brother, it wasn’t always easy to do around my son, right? I have some regrets about that today. We should have just included him all the way. But you don’t want a little child to see his uncle in such a state, sleeping by the dinner table.

Leon’s story about the difficulties regarding including his brother in the family showed an example of the “addict uncle” as overshadowing the “brother” and, as such, constructing a troubled position both for Leon and his brother. When Leon’s brother fell asleep by the dinner table, he departed from how an “uncle” should behave. Then, Leon had to let his responsibility as a “parent” overshadow his responsibility as a “brother” by showing consideration for his children before his brother. Still, Leon’s story also exposed his care for his brother by wanting to hide his position as an “addict” in order to maintain his brother’s position as his son’s “uncle.” Leon’s experiences of regrets were a consequence of not having included his brother more, even though he justified his actions by demonstrating how his duty as a “parent” in protecting his son had to be prioritized in that situation.

## Discussion

The findings show how the bereaved participants categorized their siblings as “addicts,” but also as “brothers,” “uncles,” and “unique” to make meaning of their sibling relationship. The findings display how the subject position as an “addict” is experienced as a stigmatized and troubled position. Still, when “addict” intersects with the other categories, less troubled positions can be produced. Nevertheless, these intersections can also produce other troubled positions and consequences, as discussed below.

### Mending the sibling’s legacy and protecting the family

The findings show several examples of how the participants use other and more accepted categories, such as “brother,” “uncle,” or “unique,” and, as such, avoid the troubled position of an “addict.” Similar characterizations are found in Perrin’s (2022) study, where she describes that many of her informants emphasized their siblings as “not a drug addict” (p. 208). Although it was evident in her work that the informants’ deceased siblings had suffered from PSU and had several problems caused by their drug use, the informants emphasized that their deceased siblings did not fit into the stereotypical understanding of a “drug addict.” The way the siblings in the present paper categorize their deceased as “unique” rather than “addicts” may be understood as a way of keeping their good memories and constructing a culturally acceptable legacy. This corresponds with a trend also identified in Perrin’s (2022) analysis, wherein she describes a tendency among the interviewed siblings to focus on preserving the uniqueness of the relationship rather than on the negative aspects surrounding a DRD.

The use of normative acceptable characteristics can also be understood as a strategy to produce less stigmatized positions and, as such, a way to protect oneself from stigma that might be related to the “addict” position. Perrin (2022) argues that explaining a sibling’s DRD to others is more complex than when someone dies from less stigmatized causes. The loss of a close person to a DRD has been characterized as stigmatized bereavement (Stout & Fleury-Steiner, 2023), and stigma has shown to be both societal (Dyregrov & Selseng, 2021; Reime et al., 2024) and self-induced (Stout & Fleury-Steiner, 2023), causing a silence surrounding the death both from the bereaved individuals but also from supporters (Titlestad et al., 2021). Kheibari et al. (2022) found that people were more likely to characterize people who died by overdoses with more negative wordings, such as “pathetic,” “an embarrassment,” “irresponsible,” and “stupid,” compared to those who died by suicide. Bottomley et al. (2023) argue that such attitudes are likely to be internalized by close relatives and contribute to their experiences of shame and stigma. Such self-inflicted stigma may be one explanation for why the participants in both Perrin’s (2022) work and the present study portrayed their deceased siblings as “unique.”

When siblings categorize their deceased siblings with less troubled positions, this can make it easier to talk about death and grief and, as such, create opportunities for social support in bereavement. By portraying their siblings with normative acceptable categories, the siblings can overcome the barriers to support described in other studies related to taboos and their own social withdrawal. For example, Lindeman, Lorås, et al. (2023) show how relatives of an individual with PSU felt that others could not understand their situation and found themselves unable to seek help or talk to others about their problems related to their family member’s PSU. Such withdrawal from others is also documented in other works exploring stigmatized bereavement (Azorina et al., 2019; Titlestad et al., 2021) and is, among others, related to a need to protect oneself from further harm to one’s self-identity (de Hooge et al., 2010). The bereaved siblings in Perrin’s (2022) study describe an ambivalence about whether the circumstances surrounding a sibling’s DRD should be made public. When the cause of the death was kept private, it was explained in Perrin’s (2022) research as a strategy to protect the family from other people’s harsh judgements as well as facilitating possibilities for support from their social networks. The intermingling of the category of “addicts” with other ones also points to the stigma regarding DRDs that exists in society, which can make this meaning-making necessary.

### Recognizing siblings as both close and troubled

The participants in the present study drew on common assumptions about sibling bonds as solid relationships, with mutual responsibilities of caring and being there for each other, portrayals also confirmed in research. For example, in the paper by Gulløv et al. (2021), sibling relationships are described as feelings of having someone who is “always there for you” and “who takes an interest in your life and backs you” (p. 14), while Chua (2020) describes the loss of a sibling as losing a part of oneself. Nevertheless, several of the participants in the present work also perceived their “addict” brothers or sisters as not reliable and causing them disappointments. Thus, when the “addict” siblings’ actions deviated from the normative expectations of “siblings,” their positions became troubled. The participant’s alteration between depicting their relationship with the deceased sibling as close and as troubled can be interpreted as ambivalence.

In the present study, many of the portrayals of close and solid sibling relationships were related to either the time before the sibling developed PSU, or to periods where they did not use drugs. The troubled characterizations of sibling relationships were mainly related to the period after the siblings had developed PSU. Such changes that occur in the quality of a relationship when a sibling develops PSU, as shown in the present work, adhere to Tsamparli and Frrokaj (2016) research displaying how PSU represented a radical change in the quality of the sibling relationship, causing feelings of shame and embarrassment among the siblings who did not have PSU. Lindeman, Lorås, et al. (2023) report siblings describing their brothers and sisters with PSU with negative behavioural characteristics, such as becoming less reliable, unpredictable, and violent.

The lack of cultural recognition of the ambivalence that siblings of persons with PSU endure when their brothers or sisters differ from the normative expectation of a “sibling” can explain the use of repairing categories, as shown in this work, to make their sibling relationship legitimate. Greif and Woolley (2016) claim that although negative feelings towards a sibling may be part of the sibling relationship in childhood, such mixed emotions are not as easy to acknowledge in adulthood. Commonly, the siblings of those struggling with PSU will experience an ambivalence in their relationship, wanting closeness and trust with their brother or sister, while they also need physical and emotional distance (Greif & Woolley, 2016). While Greif and Woolley (2016) emphasize how the siblings of those struggling with PSU often suppress their feelings in these situations, the present study has, from an intersectional perspective, revealed how the siblings make efforts to give meaning to this ambivalence by the usage of repairing strategies. In bereavement, meaning-making is perceived as essential to come to terms with the loss and for a healthy bereavement process (Neimeyer et al., 2014). The siblings’ attempts to repair the legacy of their deceased sibling can, therefore, also be understood as an essential strategy in their grief work.

### Categorizations causing regret and self-blame

The feelings of self-blame and regret described by some of the participants in the present study are common reactions among family members in cases of drug death bereavement that have been discussed in several works (O’Callaghan et al., 2023; Titlestad et al., 2021) and, among other aspects, have been related to the “preventable nature” of DRDs (Lambert et al., 2022). Perrin (2022) shows how the bereaved siblings experience remorse for not having done enough for their siblings when they were alive, feelings which intensified when their siblings died, and may be related to self-blame as an inter-psychological phenomenon. However, this paper gives insight into how dominant moral conceptions of drug problems and sibling relationships in the sociocultural context may produce self-blame.

Meaning-making can contribute to producing troubled positions of blame that are dependent upon their categorizing of themselves and their siblings with PSU, as highlighted in the present study. A dominant discourse in society depicts problematic drug use as caused by adversities in the family, and as such, blames the family for the person’s PSU or DRD (Hanninen & Koski‐Jannes, 1999; Sibley et al., 2020). If this discursive understanding is taken up by the siblings or their environment, it can exaggerate feelings of self-blame and the need to protect the family’s reputation. In addition, regret and self-blame can be produced by a culturally dominant understanding of siblings, identified in this work as a joint responsibility of caring and being responsible for each other. This expectation towards siblings’ relationships adheres to findings in Tsamparli and Frrokaj (2016) research, wherein many of the siblings without PSU took a parental role towards their siblings with PSU, whom they described as “sensitive” and “vulnerable” (p. 139).

Another trigger for self-blame and regret shown in the present work is the siblings’ prioritizing of their parental responsibilities before their roles as “siblings.” This finding can be understood in light of the dominance of the parent perspective demonstrated in studies of bereavement, which describe how the bereaved siblings experience their parents as more important grievers than themselves (Davidson, 2018; Lindeman, Lorås, et al., 2023; Perrin, 2022). In this paper, the participants’ choice to prioritize their children before their brothers or sisters was justified by their categorization as “parents,” which, in addition to parents’ natural biological and juridical responsibility for their children, confirms the understanding of a hierarchical family order, which ranks a parent’s concern for their children on top (Peskin, 2019; Robson & Walter, 2013). Despite being used as a legitimating category when the siblings gave meaning to their priorities, the category of “parent” also stood out as a troubled position for the siblings. By overshadowing the “sibling” position, the “parent” role produced negative feelings for not fulfilling the cultural expectations of the “sibling” one.

Finally, the findings from the present study suggest that differences related to birth order might affect the degree to which the siblings talk about their responsibilities, and hence their experiences of self-blame or regret if they are not able to fulfil these expectations. Gulløv et al. (2021) highlight the importance of examining the dynamics of positioning based on birth order when studying how siblings are categorized. In Perrin’s (2022, p. 293) work, the older siblings with PSU were portrayed as “heroes and protectors, who made younger family members feel loved and safe,” and many of the older siblings experienced guilt, frustration, and regret for having failed to protect their younger sibling from developing PSU (Perrin, 2022). This aspect of categorization is only displayed briefly in this paper. However, the identified dynamics between siblings based on birth order seem to be of importance for understanding how many of the bereaved siblings make meaning of the relationship to the sibling who is deceased from a DRD and may be considered a topic in need of further investigation.

## Conclusion and implications for practice

The present study gives insight into how bereaved siblings, after a DRD, categorize the relationship they had with their deceased brother or sister to give meaning to it. The categories are also linked to the norms at stake in everyday situations and display classifications based on the characteristics, responsibilities, and dynamics used to portray these relationships. The stereotypical understanding of an “addict” is found to be a troubled subject position and does not adhere to how the participants in this work give meaning to the relationship they had with their deceased brothers and sisters. As such, the siblings overshadow the troubled position by categorizing their deceased brothers or sisters in more legitimate categories, creating opportunities for the validation of their loss and, thus, prospects of social support from their social surroundings. The intersection between “addict” and other repairing categorizations gives insight into the complexity of sibling relationships in this context. The analysis shows how the bereaved siblings actively engage in meaning-making to produce less troubled positions for the deceased individual, as well as themselves and their families.

This article provides practitioners, bereaved families, and their social networks with an awareness of how bereaved siblings may be affected by categorization processes concerning both their deceased siblings with PSU as well as themselves. Such knowledge can be used to reflect on how social categories construct positions of that are troubled to varying extents and can contribute to increased awareness among practitioners and social networks on how to help and support bereaved siblings after DRDs in their meaning-making.
